# Relationship between occupational burnout and demographic variables among nurses in Jahrom, Iran

**DOI:** 10.11604/pamj.2019.34.22.15642

**Published:** 2019-09-11

**Authors:** Shahrzad Yektatalab, Khadijeh Honarmandnejad, Roksana Janghorban

**Affiliations:** 1 Department of Nursing, School of Nursing and Midwifery, Community Based Psychiatric Care Research Center, Shiraz University of Medical Sciences, Shiraz, Iran; 2 Department of Nursing, School of Nursing and Midwifery, Student Research Committee, Shiraz University of Medical Sciences, Shiraz, Iran; 3 Department of Midwifery, School of Nursing and Midwifery, Community Based Psychiatric Care Research Center, Shiraz University of Medical Sciences, Shiraz, Iran

**Keywords:** Nurses, occupational burnout, demographic factors

## Abstract

**Introduction:**

Occupational burnout is a psychological syndrome caused by the accumulation of work-related stress and characterized by intolerance, high levels of emotional exhaustion, depersonalization, and the perception of low personal accomplishment. The present study aimed to evaluate the relationship between occupational burnout and all demographic variables among the nurses in Jahrom, Iran.

**Methods:**

The present descriptive-analytical study was carried out during 2016 among nurses employed at Motahari Hospital and Peymanieh Hospital, both affiliated to Jahrom University of Medical Sciences (Iran). Based on the inclusion criteria, a total of 250 participants were selected. The data collection instruments included a demographic questionnaire and the Maslach burnout inventory. The data were analyzed using the SPSS statistical software (version 16.0) by descriptive statistics and Spearman's test.

**Results:**

Among the participants, 223(89.2%) nurses suffered from a moderate to high level of occupational burnout. There was a significant correlation between personal accomplishment and age (r=0.21, P=0.002) and education level (r=-0.16, P=0.01). Additionally, income level had a significant correlation with emotional exhaustion (r=-0.38, P=0.001), depersonalization (r=-0.3, P=0.001), and personal accomplishment (r=0.35, P=0.001). A significant relationship was also found between sex and depersonalization (r=-0.15, P=0.02). However, there was no significant relationship between occupational burnout subscales and the number of children, type of hospital ward, type of employment, and marital status (P>0.05).

**Conclusion:**

A significant positive correlation was found between the subscales of occupational burnout and younger age, low income, high education, and male nurses.

## Introduction

Occupational burnout (OB) is a psychological syndrome caused by the accumulation of work-related stress and characterized by intolerance, high levels of emotional exhaustion, depersonalization, and the perception of low personal accomplishment. OB can adversely affect the physical and mental health, performance at work, and even lead to loss of valuable workforce. It occurs in any profession, but particularly among health care workers due to the heavy workload [1-3]. It is estimated that OB in health care centers has been the cause of 30% workers' illnesses and absence from work; at an annual cost of 300-400 million US dollars. Various studies have reported that among all health professionals, nurses are at a higher risk of OB [4,5]. The incidence of OB has been estimated at a staggering level of >40% among professional nurses [6], particularly emergency room nurses, as they are daily exposed to the suffering and discomfort of patients as well as death. Additionally, they suffer from insomnia, stress and anxiety due to the working conditions in hospitals, or from working in a negative competitive workplace. Anxiety among nurses not only negatively affects their relationship with patients, but is also one of the reasons for the reduced efficiency of the health care system [7]. Studies have indicated that positive experiences in the workplace enhance professional commitment and reduce occupational stress, while negative perceptions and long-term workplace stresses result in nurse burnout [8]. A previous study has reported that the prevalence of OB was significantly related to early life stress, living alone, not having children, and not using coping strategies against problems [9]. Another study reported that high levels of personal accomplishment could improve the physical and mental well-being of nurses and encourage them to provide high-quality care to patients [10]. In light of the above, a number of studies have been conducted to evaluate the prevalence of nurse burnout and to recommend measures to prevent its occurrence. A study among Spanish nurses reported that OB was significantly correlated with demographic and personality-related variables [4]. They recommended the need for more information gathering about nurse burnout prevalence and its risk factors to determine the best intervention method to prevent this phenomenon. Studies conducted in different countries have reported a significant difference among Irish, Greek, Italian, Polish, and Dutch nurses with respect to emotional exhaustion and depersonalization [3,11]. The difference was attributed to dissimilarities between the professional environment and the role nurses play in various health care systems. Some related studies have also been conducted on this topic in Iran, however, they did not include all relevant demographic variables affecting the occurrence of nurse burnout [12,13]. To complement previous studies, we aimed to evaluate the relationship between OB and demographic variables among the nurses in Jahrom, Iran.

## Methods

**Participants:** the present descriptive-analytical study was carried out during 2016 among nurses employed at Motahari Hospital and Peymanieh Hospital, both affiliated to Jahrom University of Medical Sciences (Jahrom, Iran). The participants were selected based on the census sampling method among 266 qualified nurses from both hospitals. The inclusion criteria were a minimum of 2 years working experience, willingness to participate, and higher education. The exclusion criterion was partial completion of the questionnaires. Based on these criteria, a total of 250 participants were selected. The participants were informed about the goals of the research, methodology, and confidentiality of any disclosed information. A written informed consent was obtained from all the participants.

**Data collection:** the data collection instruments included a demographic questionnaire and the Maslach Burnout Inventory (MBI). Demographic characteristics of the participants included age, sex, level of education, marital status, number of children, job tenure, type of ward, employment status, and the presence of any mental or physical illnesses. MBI is the most common tool to measure occupational burnout. In this study, the intensity scale of MBI was used. It consisted of 22 propositions and measured three aspects of occupational burnout. Accordingly, nine propositions (1, 2, 3, 6, 8, 13, 14, 16, 20) determined emotional exhaustion, five propositions (5, 10, 11, 15, 22) determined depersonalization, and eight propositions (4, 7, 9, 12, 17, 18, 19, 21) determined personal accomplishment. The propositions were scored on a 7-point Likert scale ranging from 0 (never) to 6 (every day). Accordingly, the scores were categorized into low, moderate, and high groups. The degrees of occupational burnout subscales have been presented in Table 1 [2]. Occupational burnout was defined by scores 17 or higher in emotional exhaustion, scores 7 or higher in depersonalization, or scores 38 or lower in personal accomplishment [2]. According to Maslach and Jackson, the internal consistency of the questionnaire for emotional exhaustion, depersonalization, and personal accomplishment was 0.9, 0.79, and 0.71, respectively [2]. Furthermore, the Cronbach's alpha of the questionnaire ranged between 0.71-0.90. The test-retest reliability of the questionnaire, with a one-month interval, ranged between 0.60-0.80. The reliability of the instrument for the total scale, emotional exhaustion, depersonalization, and personal accomplishment was confirmed by Cronbach's alpha equal to 0.82, 0.80, 0.78, and 0.84, respectively [14]. Moreover, the validity of the Persian version of the MBI questionnaire was similar to that of the original version [15].

**Table 1:** degrees of occupational burnout subscales

Degree	Subscales		
	Personal accomplishment	Depersonalization	Emotional exhaustion
**0-31**	13 and above	27 and above	High
**32-38**	7-12	17-26	Moderate
**39 and above**	0-6	0-16	Low

**Statistical analysis:** the data were analyzed using the SPSS statistical software (version 16.0). Descriptive data were used to report the frequency and the Pearson correlation coefficient was used to determine the correlation between nurse burnout and demographic variables.

## Results

A total of 250 (93.98%) participants fully completed the questionnaires. Most participants were female (78.5%) aged 30-35 years. The mean age of the participants with and without burnout was 33.84±5.49 and 38.20±7.07 years, respectively. The majority were married (78%) and mainly had a B.Sc. degree in nursing (88.8%). In terms of employment, the participants had a full-time (52.5%), part-time and semi-formal (19.3%) or contractual (28%) job. Details on demographic characteristics of the participants are described in Table 2. The total mean score of the OB scale was 47.05±18.31 and the mean score for its subscales (emotional exhaustion, depersonalization, and personal accomplishment) was 20.98±11.53, 6.90±5.28, and 29.02±6.63, respectively. Among the participants, 223(89.2%) nurses suffered from a moderate to high level of OB. A total of 105 (47.1%) nurses worked at Motahari Hospital and 118 (52.9%) at Peymanieh Hospital. The intensity of OB among the participants is illustrated in Table 3. The correlation between OB subscales and demographic variables are shown in Table 4. There was a significant correlation between personal accomplishment and age (r=0.21, P=0.002). Younger age was positively correlated with personal accomplishment such that an increase in age resulted in improved personal accomplishment. The level of income was also significantly correlated with emotional exhaustion (r=-0.38, P=0.001), depersonalization (r=-0.3, P=0.001), and personal accomplishment (r=0.35, P=0.001). This implied that a decrease in the level of income resulted in higher emotional exhaustion and depersonalization and lower personal accomplishment. There was also a significant correlation between personal accomplishment and education level (r=-0.16, P=0.01). An increase in the level of education was positively correlated with personal accomplishment. Nurses with higher level of education experienced lower personal accomplishment score. However, no significant relationships were observed between the three subscales of occupational burnout and number of children. The results also indicated a significant relationship between sex and depersonalization (r=-0.15, P=0.02). Male nurses positively correlated with depersonalization and experienced a higher level of depersonalization compared to female nurses. There was no significant relationship between OB subscales and the number of children, type of hospital ward, type of employment, and marital status (P>0.05).

**Table 2:** distribution of the demographic characteristics of the participants with respect to occupational burnout

Variable	Category	With burnout n=223	Without burnout n=27
		N (%)	N (%)
Gender	Female	175 (78.5)	22 (83.33)
	Male	48 (21.5)	5 (16.67)
Marital status	Married	174 (78)	20 (74.07)
	Single	45 (20.3)	7 (25.9)
	Divorced	4 (1.7)	0 (0)
Number of children	0	82(36.7)	7 (25.9)
	1	72 (32.2)	5 (18.5)
	2	62 (28.18)	15 (55.6)
	3	6 (2.73)	0 (0)
	4	1 (0.47)	0 (0)
Education level	Associate degree	14 (6.27)	4 (14.81)
	Bachelor’s degree	198 (88.7)	22 (81.48)
	Master’s degree	11 (4.93)	1 (3.7)
Ward	Intensive care	127 (56.95)	15 (55.55)
	General	96 (43.04)	12 (44.44)
Employment status	Permanent full-time	117 (52.46)	18 (66.6)
	Permanent part-time	43 (19.28)	2 (7.4)
	Semi-formal	37 (16.59)	5 (18.5)
	Contractual	26 (11.65)	2 (7.4)

**Table 3:** distribution of the participants with respect to occupational burnout subscales degrees

Occupational burnout degree	Burnout subscales		
	Emotional exhaustion N (%)	Depersonalization N (%)	Personal accomplishment N (%)
Low	115 (46)	152 (60.8)	58 (23.2)
Moderate	72 (28.8)	67 (26.8)	62 (24.8)
High	63 (25.2)	31 (12.4)	130 (52)

**Table 4:** The correlation between occupational burnout subscales and demographic factors

Demographic factors	Emotional exhaustion		Depersonalization		Personal accomplishment	
	r	‡P-value	r	‡P-value	r	‡P-value
Age	-0.08	0.18	-0.11	0.08	0.21	0.002
Income level	-0.38	0.001	-0.3	0.001	0.35	0.001
Number of children	-0.06	0.34	-0.07	0.3	0.11	0.08
Education level	0.09	0.16	0.12	0.06	-0.16	0.01
Gender	-0.005	0.94	-0.15	0.02	-0.04	0.51
Type of ward	-0.1	0.1	-0.12	0.07	0.007	0.92
Marital status	0.09	0.14	-0.008	0.91	-0.01	0.78
Employment status	-0.009	0.89	-0.07	0.28	-0.02	0.7

‡ Spearman

## Discussion

The demographic characteristics of the participants in the present study were comparable to those in Kravits *et al*. study [16]. The majority of the participants were aged 30-34 years and 75% of them had a permanent contract or contractual job. In parallel to having a job, they were also housewives which resulted in excessive stress and anxiety and subsequently affected their mental health [17]. Zanganeh *et al*. investigated the relationship between OB and the general health of nurses in two Iranian cities (Abadan and Khorramshahr). In their study, out of the 118 participants, 88.9% were female and 9.57% were male. The majority of the participants were aged 22-30 years [18]. In Karimyar’s study, 162 out of the 212 nurses under investigation (75.5%) were female, 130(61.3%) were married, 177(83.5%) held a B.Sc. degree, and their mean age was 25-30 years [19]. In contrast to the results of the present study, the above-mentioned studies reported a lower mean OB, which might be due to the differences in the mean age of the participants. The results showed that the majority of the participants experienced a moderate to high level of OB. In line with our result, a study on OB among Indonesian nurses reported that OB was highly prevalent and its level was higher than the average among the nurses [4]. Some other studies also reported a high prevalence of OB among nurses [3,20,21]. It has been reported that nurse burnout is the result of occupational stress and that there is a strong relationship between OB, environmental stress, and personality traits [21]. Therefore, the prevalence of OB could be the result of being faced with job-related stress as well as factors such as role ambiguity, role conflict, work pressure, and inadequate workplace conditions [20,22]. Additional causes could be due to being left out of the decision-making process, lack of accountability by officials, and lack of cooperation between nursing units and other wards [19,23]. We found that the majority of the participants had a high to moderate level of emotional exhaustion and depersonalization, and a low level of personal accomplishment. Momeni *et al*. reported a high degree of emotional exhaustion and depersonalization, and a low degree of personal accomplishment among nurses [12]. Similar results in the UK were reported by Adriaenssens *et al*. [3]. In contrast, a significantly lower level of nurse burnout was reported in Iran (Mashhad) [24], Scotland [25], and in China [26]. Such differences could be related to the working conditions and the role of nurses in various health care systems around the world. They could also be attributed to the level of independence at work, the span of control, partnership, and the level of communication between nurses in the workplace [27]. The results showed a significant relationship between personal accomplishment and age. Personal accomplishment improved with age and was associated with a lower OB score. Spooner reported a negative correlation between age and depersonalization such that younger nurses suffered a higher level of nurse burnout [28]. Momeni *et al*. also reported different depersonalization scores with respect to different age groups. Based on the above, it seems that age could have an adverse effect on some OB subscales. Since the ability to handle occupational issues improved with higher age and experience, therefore, more experience could be associated with effective solving of unpredictable situations [12].

In line with Cañadas-De la Fuente *et al*. study [4], our results also showed a significant difference between male and female nurses in terms of the mean depersonalization score. Male nurses achieved a higher score, which indicated a personality difference between male and female nurses. A statistically significant correlation was found between OB subscales and income level. Other studies also reported that nurses consider low income as an important cause of work-related stress [19, 29]. Anderson and Brooks also reported that only 57% of the nurses were satisfied with their income level [22]. Consequently, low income level could be considered as a cause of nurse burnout. In line with the findings of Lin *et al*. [30], we also found no statistically significant difference between marital status and OB subscales. However, it was observed that married female nurses experienced a higher level of emotional exhaustion due to additional responsibilities as a housewife. On the other hand, they had a sense of higher personal accomplishment due to family support. In contrast, another study reported a relationship between marital status and emotional exhaustion and stated that marital status was the predictor of at least one OB subscales [4]. The difference between these results might be due to different sample sizes. Moreover, it seems that emotional exhaustion could also be associated with other variables such as personality traits. In a study by Abarghouei *et al* they concluded that the stress resulting from the loss of job by some employees had a ripple effect on others. In other words, not only the employees who had lost their jobs were stressed, but those who witnessed the event were also stressed due to the concern about their own job security [5]. In contrast, we found no significant relationship between OB subscales and employment status. This could be due to the fact that the Iranian insurance system provides equal support to people irrespective of the employment status. Therefore, employment status did not affect OB. The results of the present study showed no significant relationships between OB subscales and working in different hospital wards (general ward or intensive care unit). Hooper *et al*. reported that about 82% of nurses working in the emergency wards had a moderate to high level of nurse burnout. However, they found no significant difference between the nurses on emergency wards and those on oncology, nephrology, and special wards in terms of compassion satisfaction, job burnout, and compassion fatigue [31]. Irregularities, unpredictable situations, stressful conditions, lack of control, a limited time frame for assessing the effect of therapeutic interventions on the patients, and frequent contact with patients with pain and anxiety and stressful conditions have also been considered to be stressful issues for nurses working in emergency departments [32]. In the same vein, Cabera *et al*. found that working on a specific type of ward was associated with nurse burnout, which is in contrast to the present study [33]. The difference in the results could be due to the way we categorized the wards since we considered the emergency ward, Cardiac/Coronary Care Unit (CCU), Intensive Care Unit (ICU), and dialysis as intensive care units while other units were grouped as the general ward. The results showed a significant relationship between personal accomplishment and education level. Similarly, Talaei *et al*. reported that nurses with higher education levels experienced more nurse burnout in terms of personal accomplishment [24]. However, Qu *et al*. found that Chinese nurses with basic education scored a significantly higher OB level than those with an intermediate or advanced education [34]. Different employment policies could be the reason behind such a difference in the results. Iranian nurses with a higher level of education are given a variety of tasks, responsibilities, and accountability compared to nurses with lower education. However, their salaries were not much different from each other. The main strength of the present study is the inclusion of the majority of demographic variables and the use of the MBI questionnaire to evaluate OB and its subscales. However, the small sample size was the main limitation which in turn did not allow the generalizability of the findings.

## Conclusion

The results of the present study showed a high prevalence of OB among the nurses in Jahrom, Iran. Furthermore, OB subscales were significantly correlated with age, income level, sex, and education level. Younger age, low income, high education, and male nurses were positively correlated with OB subscales. Identification of those factors affecting nurse burnout plays an important role in preventing this phenomenon among nurses.

### What is known about this topic

- Nurses experience occupational burnout;
- Some demographic factors affect nurses’ occupational burnout.

### What this study adds

- Increase in age may be effective in decreasing depersonalization and enhancing personal accomplishment among nurses;
- Nurses with higher education levels may experience higher occupational burnout.

## Competing interests

The authors declare no competing interests.

## References

[1] Salvagioni DAJ, Melanda FN, Mesas AE, Gonzalez AD, Gabani FL, Andrade SMd (2017). Physical, psychological and occupational consequences of job burnout: A systematic review of prospective studies. PLoS ONE.

[2] Maslash C, Jackson S, Leiter M (1996). Maslach Burnout Inventory manual.

[3] Adriaenssens J, De Gucht V, Maes S (2015). Determinants and prevalence of burnout in emergency nurses: a systematic review of 25 years of research. Int J Nurs Stud.

[4] Cañadas-De la Fuente GA, Vargas C, San Luis C, García I, Cañadas GR, De la Fuente EI (2015). Risk factors and prevalence of burnout syndrome in the nursing profession. Int J Nurs Stud.

[5] Abarghouei MR, Sorbi MH, Abarghouei M, Bidaki R, Yazdanpoor S (2016). A study of job stress and burnout and related factors in the hospital personnel of Iran. Electron Physician.

[6] Brand S, Beck J, Hatzinger M, Harbaugh A, Ruch W, Holsboer-Trachsler E (2010). Associations between satisfaction with life, burnout-related emotional and physical exhaustion, and sleep complaints. World J Biol Psychiatry.

[7] Antai-Otong D (2009). Psychiatric nursing: Biological and behavioral concepts.

[8] Ahola K, Toppinen-Tanner S, Huuhtanen P, Koskinen A, Väänänen A (2009). Occupational burnout and chronic work disability: an eight-year cohort study on pensioning among Finnish forest industry workers. J Affect Disord.

[9] Pereira SDS, Teixeira CAB, Reisdorfer E, Gherardi-Donato ECDS, Juruena MF, Cardoso L (2015). Burnout in nursing professionals: Associations with early stress. British Journal of Mental Health Nursing.

[10] Nayeri ND, Negarandeh R, Vaismoradi M, Ahmadi F, Faghihzadeh S (2009). Burnout and productivity among Iranian nurses. Nurs Health Sci.

[11] O’Mahony N (2011). Nurse burnout and the working environment. Emerg Nurs.

[12] Momeni H, Salehi A, Seraji A (2010). The comparison of burnout in nurses working in clinical and educational sections of Arak University of Medical Sciences in 2008. Arak University of Medical Sciences Journal.

[13] Aghajani M (2013). The Professional Burnout of Nurses in Different Wards. Journal of Research Development in Nursing and Midwifery.

[14] Mehafarid M, Khakpour M, Jajarmi M, Alizadeh mousavi A (2015). Effectiveness of positive thinking training on hardiness and resilience and Job burnout in women nurses. Journal of Nursing Education.

[15] guayo R, Vargas C, de la Fuente EI, Lozano LM (2011). A meta-analytic reliability generalization study of the Maslach Burnout Inventory. Int J Clin Health Psychol.

[16] Kravits K, McAllister-Black R, Grant M, Krik C (2010). Self-care strategies for nurses: A psycho-educational intervention for stress reduction and the prevention of burnout. Appl Nurs Res.

[17] Abdi Masooleh F, Kaviani H, Khaghanizade M, Momeni Araghi A (2007). The relationship between burnout and mental health among nurses. Tehran Univ Med J.

[18] Zanganeh S, Moradbeygi K, Rasteh M, Raisifar Z (2014). Investigating the Degree of Job Burnout and Its Relation with the General Health of Nurses Working in Hospitals of Abadan and Khoramshahr in 2012. Scientific Journal of Ilam University of Medical Sciences.

[19] Karimyar Jahromi M (2014). The etiology of burnout syndrome and the levels of stress among nurses. Journal of Jahrom University Medical Science.

[20] Toh SG, Ang E, Devi MK (2012). Systematic review on the relationship between the nursing shortage and job satisfaction, stress and burnout levels among nurses in oncology/haematology settings. Int J Evid Based Healthc.

[21] Lorenz V, Benatti M, Sabino M (2010). Burnout and Stress Among Nurses in a University Tertiary Hospital. Rev Lat Am de Enfermagem.

[22] Aziz nejad P, Hosseni S (2006). Occupational burnout and its causes among practicing nurses in hospitals affiliated to Babol University of Medical Sciences, 2004. Journal of Babol University Medical Sciences.

[23] Brooks BA, Anderson MA (2006). Nursing work life in acute care. J Nurs Care Qual.

[24] Talaei A, Mokhber N, Mohammad-Nejad M, Samari AA (2009). Burnout and its related factors in staffs of university hospitals in Mashhad in 2006. koomesh.

[25] Kilfedder CJ, Power KG, Wells TJ (2001). Burnout in psychiatric nursing. J Adv Nurs.

[26] Wu S, Zhu W, Wang Z, Wang M, Lan Y (2007). Relationship between burnout and occupational stress among nurses in China. J Adv Nurs.

[27] McGonagle AK, Barnes-Farell JL, Di Milia L, Fischer FM, Hobbs BBB, Iskra-Golec I (2014). Demands, resources, and work ability: a crossnational examination of health care workers. European Journal of Work and Organizational Psychology.

[28] Spooner-Lane R (2004). The Influence of Work Stress and Work Support on Burnout in Public Hospital Nurses.

[29] Tsai Y, Liu CH (2012). Factors and symptoms associated with work stress and health-promoting lifestyles among hospital staff: a pilot study in Taiwan. BMC Health Serv Res.

[30] Lin F, St John W, McVeigh C (2009). Burnout among hospital nurses in China. J Nurs Manag.

[31] Hooper C, Craig J, Janvrin DR, Wetsel MA, Reimels E (2010). Compassion satisfaction, burnout, and compassion fatigue among emergency nurses compared with nurses in other selected inpatient specialties. J Emerg Nurs.

[32] Kwon KJ, Lee SH (2012). Occupational stress and coping styles as factors affecting the burnout of clinical nurses. J Korean Acad Nurs Adm.

[33] Cabera Gutierres L, Rojas P, salinas Tovar S (2005). Burnout Syndrom among Mexican hospital Nursery staff. Rev med inst Mex Seguro Soc.

[34] Qu HY, Wang CM (2015). Study on the relationships between nurses' job burnout and subjective well-being. Chinese Nursing Research.

